# Non-Nodal CD5-Negative Mantle Cell Lymphoma with Secondary TP53 Deletion

**DOI:** 10.1155/2020/9185432

**Published:** 2020-03-18

**Authors:** Robert K. McCall, Changlee S. Pang, Mark J. Pettenati

**Affiliations:** ^1^Molecular Pathology Laboratory Network, Maryville, TN, USA; ^2^University of Colorado School of Medicine, Aurora, CO, USA; ^3^Wake Forest Baptist Medical Center, Winston-Salem, NC, USA

## Abstract

Mantle cell lymphoma is a non-Hodgkin lymphoproliferative neoplasm with several clinical and morphologic variants linked, primarily, through genetic derangement of the *cyclin D1* locus. Aberrant phenotypes have been described, though prognostic data in such cohorts are limited due to a paucity of cases. We report a case of mantle cell lymphoma with non-nodal clinical presentation, aberrant loss of CD5 expression, and concomitant cytogenetic deletion of 17p. While non-nodal disease is often associated with an improved prognosis in mantle cell lymphoma, this 67-year-old patient experienced a more challenging clinical course with a poor initial response to chemotherapy. Therefore, this case may represent a type of non-nodal mantle cell lymphoma with a prognosis similar to that of classical cases due to the additional phenotypic and genetic alterations found in this patient.

## 1. Introduction

Mantle cell lymphoma (MCL) is a distinct lymphoproliferative disorder requiring the combination of morphologic, immunophenotypic, and cytogenetic information for diagnosis. Classically, MCL is characterized by infiltration, either nodular or diffuse, of lymphoid tissue by small-to-medium-sized B-lymphocytes with irregular nuclei and scant cytoplasm [1, 2]. Immunohistochemical staining is typically positive for CD5, CD19, CD20, and CD79b. CD5 is especially helpful in differentiating between MCL and low-grade lymphomas such as follicular lymphoma or marginal zone lymphoma, although, multiple CD5-negative cases of MCL [3–5] have been reported in the past few decades. Derangement of the *cyclin D1 (CCND1)* locus has proved more diagnostically sensitive than CD5 positivity for the identification of MCL [3, 5] with t(11; 14)(q13; q32) representing the most common cytogenetic aberration. A wide range of secondary nonspecific genetic abnormalities has also been shown to occur in cases of MCL [6, 7]. For example, *TP53* mutation/17p deletion portends a poor prognosis according to several studies [8–15]. To complicate matters, morphologic variants of MCL include blastoid, pleomorphic, marginal zone-like, and small cell types. Clinical heterogeneity has also been established with variable involvement of the lymph nodes, spleen, and bone marrow. Non-nodal MCL (a.k.a. mantle cell leukemia) often presents as asymptomatic lymphocytosis with spleen and/or bone marrow infiltration by atypical lymphocytes, similar to chronic lymphocytic leukemia/small lymphocytic lymphoma (CLL/SLL). Interestingly, all previously reported cases of non-nodal MCL [4, 15–17] have been CD5-positive.

We present a rare case of CD5-negative non-nodal MCL with secondary *TP53* mutation/17p deletion and a complicated clinical course.

## 2. Case Report

**Figure 1:**
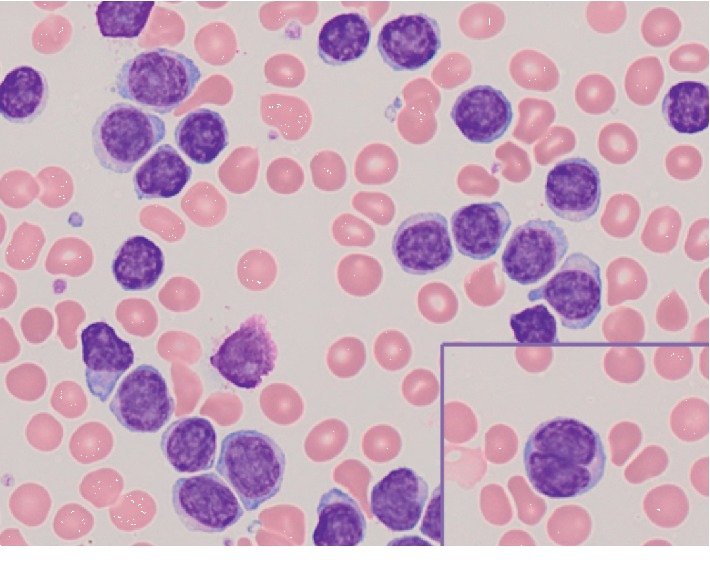
Peripheral blood smear demonstrating CLL/SLL-like morphology of atypical lymphocytes. Inset shows a representative peripheral blood lymphocyte with characteristic nuclear irregularity seen in a subset of B-cells in this case.

A 67-year-old male with a past medical history of basal cell carcinoma, hyperlipidemia, and chronic back pain, secondary to a compression fracture of the T12 vertebra status after vertebroplasty, presented with an infection of the anterior shin in addition to left upper quadrant discomfort. The patient denied fever, chills, night sweats, and weight loss. Physical examination failed to reveal palpable lymphadenopathy or hepatosplenomegaly. Laboratory testing identified a prominent leukocytosis with a white blood cell count (WBC) of 449,000, absolute lymphocyte count (ALC) of 416,400, platelet count of 120,000, and lactate dehydrogenase level of 185 U/L (reference range: 90–250 U/L). The peripheral blood smears showed numerous small-to-medium-sized lymphocytes with relatively round nuclear contours, coarsely clumped chromatin (“soccer ball” pattern), and inconspicuous nucleoli (Figure 1), all of which are features morphologically consistent with chronic lymphocytic leukemia. A PET/CT scan was performed and notable for splenomegaly (18 cm) with mild hypermetabolic activity. Scattered prominent para-aortic lymph nodes were also identified, though not enlarged or hypermetabolic.

The bone marrow biopsy material was B+ fixed, paraffin embedded, and stained with hematoxylin and eosin (H&E). Bone marrow aspirate smears and additional peripheral blood smears were stained with Wright–Giemsa. The majority of lymphocytes on the repeat peripheral blood smears were morphologically consistent with that described for the initial smear (Figure 1); however, a subset of these small lymphocytes contained focal nuclear membrane irregularities with deep nuclear grooves (Figure 1, inset). Additionally, a subpopulation of lymphocytes was noted with cells slightly increased in size and conspicuous-to-distinct, centrally located nucleoli, reminiscent of prolymphocytes. The bone marrow aspirate smears were dominated, 98% of cells, by the heterogenous lymphocyte population reflected in the peripheral blood. Neoplastic transformed cells resembling centroblasts or immunoblasts were not identified in the aspirate material. The bone marrow core biopsy and clot section were hypercellular for patient age (80%), and normal marrow elements were diffusely replaced by monomorphic atypical lymphocytes. The atypical lymphocytes were small in size and contained round to mildly irregular nuclear contours, inconspicuous nucleoli, and scant cytoplasm.

**Figure 2:**
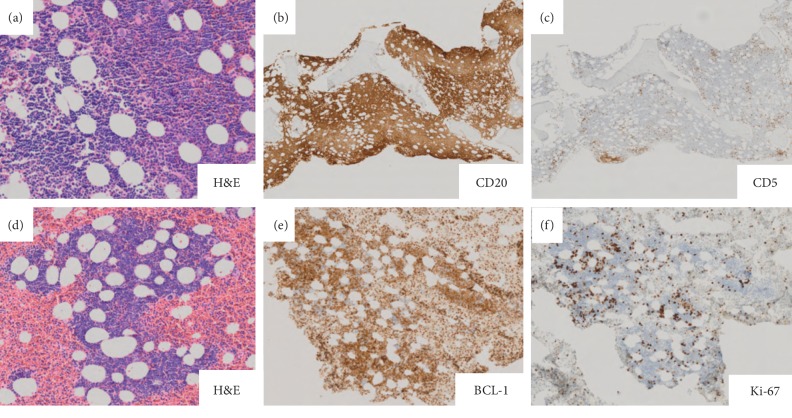
(a) H&E, high power view of bone marrow core needle biopsy with diffuse lymphoid infiltrate; (b) CD20 highlights B-cell lineage of lymphoid infiltrate; (c) CD5 expression is not seen in the neoplastic lymphoid population; (d) H&E: intermediate power view of clot section with diffuse lymphoid infiltrate; (e) BCL-1 (cyclin D1) is expressed by the neoplastic lymphoid population; (f) Ki-67 indicates a low proliferation rate in MCL.

Immunophenotype was established by flow cytometry of the initial peripheral blood sample and immunohistochemistry on the bone marrow core biopsy and clot section (Figure 2). Flow cytometric analysis revealed a monoclonal B-cell population expressing CD19, CD20 (moderate), FMC7, and kappa light chain (moderate to bright). These cells failed to express CD2, CD3, CD4, CD5, CD7, CD8, CD10, CD23, and CD38. Immunohistochemistry highlighted a diffuse population of CD20 (Figure 2(b)), CD79a, and cyclin D1-positive atypical lymphocytes (Figure 2(e)). The same cells were negative for CD5 (Figure 2(c)), CD10, CD23, and SOX11. Ki-67 (MIB1) immunohistochemistry suggested a low proliferation rate (overall 10%, Figure 2(f)).

**Figure 3:**
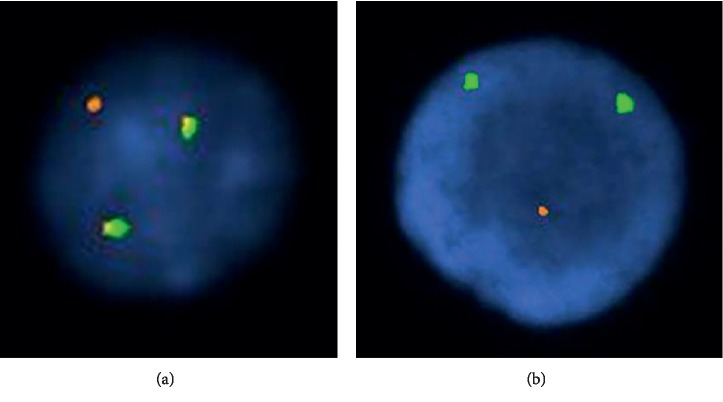
Interphase FISH demonstrating (a) t(11; 14) gene rearrangement by dual fusion signal (yellow) and (b) loss of a single copy of 17p (red/orange).

Cytogenetic studies (band resolution: 460) on the bone marrow aspirate material were interpreted as normal male karyotype with no observable clonal chromosomal abnormalities. Subsequent fluorescent *in situ* hybridization (FISH) revealed cyclin D1/immunoglobulin heavy chain (*CCND1*/*IgH*) fusion events in 66% of cells analyzed (Figure 3(a)) and deletion of the *TP53* locus (17p-) in 98% of cells analyzed (Figure 3(b)).

Initial treatment of this patient involved leukophoresis (500 cc WBC), allopurinol (300 mg/day), and induction chemotherapy with bendamustine (90 mg/m^2^ × 2). Due to an elevated WBC on day 1 of chemotherapy, the patient again underwent leukophoresis. No tumor lysis was observed, and the patient was discharged on day 4 after induction. This initial therapy was followed by four cycles of bendamustine in combination with rituximab (R-bendamustine). Response to treatment was determined via flow cytometric analysis of the patient's peripheral blood, and the therapeutic regimen changed to rituximab, etoposide, prednisone, vincristine, cyclophosphamide, and doxorubicin (R-EPOCH) due to poor results with R-bendamustine. Following four cycles of R-EPOCH, the patient's WBC had improved to 5,100 with an ALC of 800. Bone marrow transplantation was recommended, but declined by the patient. The patient completed two years of rituximab maintenance therapy (375 mg/m^2^) and is currently on surveillance with no signs of recurrent disease.

## 3. Discussion

The neoplastic cells in classical MCL are small-to-medium-sized lymphocytes with irregular nuclear contours and minimal cytoplasm. The immunophenotype is typically CD5, CD19, CD20, CD43, FMC7, surface IgM/IgD, and cyclin D1-positive. CD23 expression may be weakly positive, but is usually negative along with CD10. As the name implies, the putative normal counterpart to the neoplastic lymphocytes is the naïve pregerminal center B-cell occupying the inner mantle zone of the lymphoid follicle [1, 2]. These morphologic and immunophenotypic criteria are not entirely specific to MCL and present a formidable diagnostic dilemma. For this reason, great care should be taken in all possible cases of non-Hodgkin low-grade lymphoma to rule out MCL as the prognosis is significantly different.

Our case highlights the morphologic and immunophenotypic crossover between MCL and other low-grade lymphomas, specifically atypical CLL/SLL. Indeed, the initial peripheral blood smear in this case was more suggestive of atypical CLL than classical MCL. Given the rarity of non-nodal MCL, the clinical scenario could also easily be interpreted as most compatible with a diagnosis of CLL. While FMC7 positivity and CD23 negativity can occur in atypical CLL [18], the moderate to bright intensity of immunoglobulin light chain expression and moderate CD20 expression in this case served as clues for further investigation and raised the pathologist's suspicion for non-nodal MCL.

The most common cytogenetic aberration in MCL is t(11; 14)(q13; q32) which juxtaposes the *CCNDI* gene on chromosome 11 and the *IgH* gene on chromosome 14. Similar to the morphologic and immunophenotypic findings, this translocation is neither completely sensitive nor specific in the diagnosis of MCL [19]. Around the time that MCL emerged as a distinct entity in 1992 [2], a veritable mountain of references, only some of which are represented here [19–22], described t(11; 14) in cases of atypical CLL as well as those lymphomas previously identified as intermediately differentiated lymphocytic lymphoma (IDLL) or centrocytic lymphoma. Although IDLL and centrocytic lymphoma are now classified as MCL, cases with t(11; 14) and atypical CLL morphology still remain somewhat controversial with regard to classification. Some authors [23, 24] have suggested that all cases of atypical CLL and leukemic MCL with t(11; 14) likely represent a common disorder. Indeed, cases of atypical CLL with t(11; 14) are not recognized as a distinct entity from MCL in the current WHO classification system [1].

Conventional cytogenetic analysis is not always sufficient for the diagnosis of *CCND1/IgH* gene rearrangements, and FISH has proven valuable in the identification of these otherwise cryptic events [23]. Secondary chromosomal abnormalities in MCL [7] may include +12, +9, del (9p21), del (6q21), del (13q14), and del (17p13), to name a few. The prognosis in cases of MCL with del (17p13) has been evaluated by several groups [9, 14, 15, 25], yielding mixed results. Recently, deletion of the *TP53* locus at 17p13 has been described in a case of non-nodal MCL with poor clinical outcome [15]. More evidence exists at the molecular level where mutation of the *TP53* gene has been linked to a poor prognosis in many studies [8–14].

Our case was remarkable for both cryptic rearrangement of *CCNDI/IgH* and cryptic deletion of 17p in a patient with CD5-negative non-nodal MCL. CLL was essentially ruled out by demonstration of the t(11; 14) gene rearrangement. Among the clinical forms of MCL, non-nodal disease has demonstrated a favorable prognosis when compared to the classical type [26]. In keeping with this trend, the present case had a low proliferation rate and lacked the significant atypia often seen in the more aggressive variants of disease. However, the additional chromosomal abnormalities in this case may have contributed to a poor initial therapeutic response. It is also possible, though this remains to be seen, that CD5-negative non-nodal MCL represents a subset of MCL with a more difficult and protracted clinical course. Indeed, the bulk of CD5-negative non-nodal MCL cases described in the literature are those briefly discussed in the backdrop of larger cohorts compiled for molecular profiling studies [26, 27]. As a result, much of the information regarding prognosis and clinical course of disease in these patients is still unknown. A recent study suggests that CD5-negative cases of MCL have significantly better progression-free survival (PFS) when compared to CD5-positive cases [28]. The authors found that this improved prognosis remained despite an enrichment of non-nodal cases in the CD5-negative group. Despite these results, further studies are necessary to fully characterize the interaction between CD5 negativity and non-nodal presentation with special emphasis on the effects of both findings occurring in concert as compared to either finding being present in isolation.
